# Motivational assessment of mice using the touchscreen operant testing system: effects of dopaminergic drugs

**DOI:** 10.1007/s00213-015-4009-8

**Published:** 2015-07-10

**Authors:** Christopher J. Heath, Timothy J. Bussey, Lisa M. Saksida

**Affiliations:** Department of Psychology and MRC/Wellcome Trust Behavioural and Clinical Neuroscience Institute, University of Cambridge, Downing Street, Cambridge, CB2 3EB UK

**Keywords:** Touchscreen, Rodent, Motivation, Progressive ratio, Effort-related choice, Dopamine

## Abstract

**Rationale:**

Touchscreens are widely used to examine rodent cognition. Current paradigms require animals to view stimuli and nose poke at an appropriate touchscreen location. After responding, there is little screen interaction and, as infra-red touchscreens eliminate the need for physical contact, minimal somatosensory feedback. It is therefore unclear if touchscreens can support the vigorous, repetitive responding required in paradigms like progressive ratio (PR) for assessing motivation and effort-related choice (ERC) for assessing decision-making.

**Objectives:**

This study aims to adapt and validate PR and ERC for the rodent touchscreen.

**Methods:**

Male C57Bl/6 mice were trained until responding on PR stabilised. Amphetamine, sulpiride and raclopride were administered via the intraperitoneal route to modify performance. Mice were transferred to ERC and paradigm parameters adjusted to demonstrate behavioural modification. ERC reward preference was assessed by home cage choice analysis.

**Results:**

PR performance stabilised within seven sessions. Amphetamine (1 mg/kg) increased and raclopride (0.3 mg/kg) decreased performance by 63 and 28 %, respectively, with a 20-min injection-test interval. Sulpiride (50 mg/kg) decreased performance by 19 % following a 40-min injection-test interval. Increasing ERC operant requirements shifted responding from the operant response-dependent preferred reward towards the freely available alternative.

**Conclusions:**

Vigorous, repetitive responding is sustainable in touchscreen PR and ERC and task validation mirrors non-touchscreen versions. Thus, motivation and reward-related decision-making can be measured directly with touchscreens and can be evaluated prior to cognitive testing in the same apparatus to avoid confounding by motivational factors.

## Introduction

The touchscreen testing system provides an approach for the assessment of complex cognition in laboratory rodents using tasks very similar and in some cases identical to those used to assess cognition in humans (Bussey et al. 2012; Nithianantharajah et al. 2013). A number of paradigms have been developed for this apparatus, allowing examination of diverse aspects of cognition, including Pavlovian conditioning, pattern separation, perceptual discrimination, attention, working memory, inhibitory control, cognitive flexibility, compulsivity and impulsivity (Horner et al. 2013; Mar et al. 2013; Oomen et al. 2013). All of these tasks are carried out using the same types of stimuli, responses and reinforcers, thus facilitating comparison between assays. This equipment permits standardised, high-throughput cognitive screening with the added advantages of automation and minimal experimenter-subject interaction. In addition, computerised data collection eliminates potential experimenter bias in data analysis. This system also exclusively utilises an appetitive learning approach and aversive stimuli are explicitly avoided in all extant tasks.

All current paradigms for these systems require animals to visually interrogate stimuli presented on the touchscreen and then interact with the device only once at a single location before a task event such as reward delivery draws them to another area of the behavioural chamber, or a stimulus is displayed in a different touchscreen location (Horner et al. 2013; Mar et al. 2013; Oomen et al. 2013). In addition, with the IR touchscreens typically used in this equipment, animals do not have to physically contact the screen to register a response. This feature is desirable as it facilitates training and enables the assessment of animals with motoric impairments. However, it also minimises the somatosensory feedback provided to the animal during a response, thereby impoverishing the touchscreen as a manipulandum. It is therefore unclear whether this device is capable of supporting the vigorous, repetitive responding that can be elicited using traditional rodent levers or nose poke apertures. If such responding can be sustained by the touchscreen, the number and variety of schedules that can be implemented in the apparatus would be substantially enhanced, as would the number of behavioural domains that could be assessed using this methodology. Notable among these currently unexamined domains are motivation and reward-related decision making, which can be assessed using the progressive ratio (PR) and effort-related choice (ERC) paradigms.

Optimisation of touchscreen versions of these tasks would be of significant value as reduced motivation is a common symptom of many neurodegenerative and psychiatric disorders including Alzheimer’s disease (Cerejeira et al. 2012; Vilalta-Franch et al. 2013), Huntington’s disease (van Duijn et al. 2014) and schizophrenia (Markou et al. 2013). In the context of schizophrenia, apathy/avolition and hedonic deficits, in addition to asociality, are major components of the amotivation sub-domain of the negative symptoms experienced by sufferers of this disease (Foussias et al. 2014). Such negative symptoms, which can also include the diminished expression subdomain symptoms of flattened affect and suppression of speech (Foussias et al. 2014), have a substantial adverse influence on patient quality of life and long-term outcome and, despite significant research effort and expenditure, remain largely refractory to amelioration by current pharmacotherapies (Chue and Lalonde 2014; Dunlop and Brandon 2015). Therefore, incorporation of assays to examine part of the amotivation negative symptom sub-domain (Foussias et al. 2014) into the extant battery of touchscreen paradigms targeting many of the cognitive symptoms of this disorder will enable concurrent evaluation of rodent models and facilitate the assessment of novel therapeutics for efficacy in both the cognitive and negative symptom domains.

In the present study therefore, we assessed whether mice could demonstrate vigorous, repetitive responding at an invariant touchscreen location, in the context of the PR and ERC paradigms. The PR task (Hodos 1961) is used to assess motivation by measuring the ability of a rodent to maintain responding in order to obtain reward in the face of a sequentially increasing response requirement (Markou et al. 2013). This paradigm has been used in both rats and mice with either lever or nose poke manipulanda (Aberman et al. 1998; Bensadoun et al. 2004; Drew et al. 2007; Gourley et al. 2008; Young et al. 2011). To our knowledge, a touchscreen variant of this task has yet to be developed. To further evaluate any differences in performance detected in the PR task, the ERC paradigm (Salamone et al. 1991) can be used. In this task, rodents are required to perform cost/benefit calculations to determine whether the emission of instrumental responses to obtain a palatable reward is more favourable than the consumption of freely available but less palatable standard chow (Salamone et al. 1991; Markou et al. 2013). Typically performed using lever manipulanda in operant chambers (Koch et al. 2000; Salamone et al. 2002; Ward et al. 2012; Nunes et al. 2013) or in a barrier T-maze (Salamone et al. 1994; Walton et al. 2002, 2003, 2005; Denk et al. 2005; Pardo et al. 2012; Markou et al. 2013), to our knowledge, a touchscreen variant of this task also remains to be developed.

## Materials and methods

### Animals

Male C57Bl/6 mice (*n* = 18; Charles River Laboratories, Margate, UK) were purchased at 8–10 weeks of age and housed in groups of three in conventional cages in a humidity- and temperature-controlled housing room with a 12-h light–dark cycle (lights on 0700). Mice were left undisturbed for 6 days following arrival to acclimate before handling commenced. Cages were changed once weekly and drinking water bottles twice weekly. All animals experienced once-daily behavioural training sessions 5–7 days a week and were used in both PR and ERC studies. All procedures were conducted during the light phase of the cycle and performed in accordance with the United Kingdom Animals (Scientific Procedures) Act (1986) and the United Kingdom Animals (Scientific Procedures) Act (1986) Amendment Regulations 2012. In the course of these experiments, three animals were euthanised due to development of mild rectal prolapses.

### Food restriction and reward habituation

Animals were handled and weighed daily to establish stable free-feeding weights. Food restriction consisted of providing limited amounts of standard laboratory chow pellets (RM 3; Special Diet Services, Essex, UK) daily to each cage to maintain all animals at approximately 85–90 % of respective baseline free feeding weight. Drinking water was available to all cages ad libitum. For the two days immediately before behavioural training commenced, a small bowl containing the liquid reward (Yazoo Strawberry UHT milkshake; FrieslandCampina UK, Horsham, UK) obtained in the PR and ERC tasks was placed in each cage coincident with chow pellet delivery to minimise neophobia (Horner et al. 2013).

### Apparatus

**Fig. 1:**
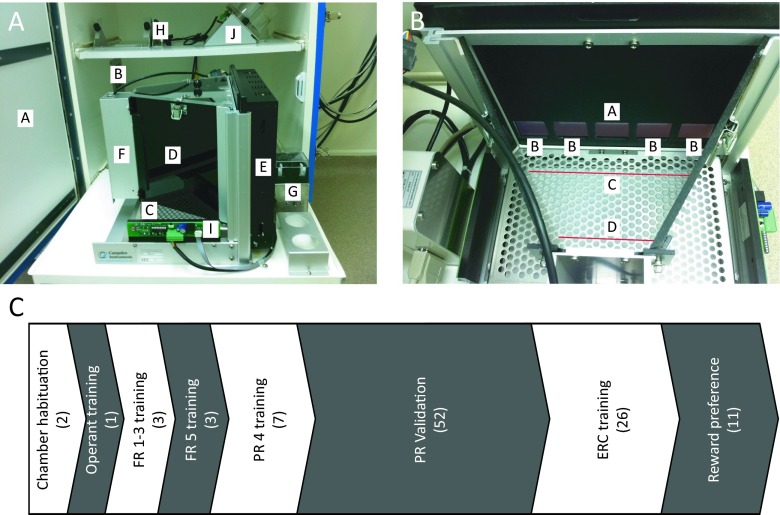
Typical touchscreen chamber and time line summary of experiments. **a** The key components of the Bussey-Saksida mouse touchscreen chamber (*A* sound-attenuating chamber; *B* ventilator fan; *C* arena floor; *D* trapezoidal arena walls; *E* touchscreen; *F* reward collection magazine; *G* liquid reward pump; *H* house light, camera, speaker and tone generator; *I* arena IR beam generator; *J* reward pellet dispenser (not used)). **b** View of arena showing ‘5-choice’ mask in position over touchscreen and position of front and rear arena IR beams (*A* central target response location; *B* non-target response location; *C* front zone IR beam; *D* rear zone IR beam). **c** Timeline summary of presented experiments. The number of days required to complete each stage is noted in *brackets*

All training was performed in standard mouse Bussey-Saksida touchscreen chambers (Campden Instruments Ltd, Loughborough, UK). These consist of operant arenas housed within dense fibreboard sound-attenuating chambers that are equipped with a fan to provide ventilation and mask background noise (Fig. 1a, b). The operant arenas consist of a perforated stainless steel floor enclosed by trapezoidal walls opening onto the touchscreen (12.1 in.; resolution 800 × 600). The touchscreen is equipped with infra-red (IR) beam arrays positioned less than 5 mm from the surface of the screen such that animals do not have to apply pressure directly to the screen for a response to be detected (Fig. 1a). A reward collection magazine connected to a liquid dispenser pump is attached to the wall opposite the touchscreen. The magazine contains an LED, which is illuminated coincident with reward delivery that in both tasks requires 800 ms pump activation to permit delivery of 20 μL of milkshake. An LED house light is provided but was not used at any stage for these tasks. In the absence of house light illumination, IR emitters allow animal observation through an IR-sensitive camera placed above the arena along with a tone generator and speaker (Fig. 1a). The chambers are also equipped with IR activity beams which run across the floor of the arena (rear beam = 3 cm from magazine port and front beam = 6 cm from screen) to monitor horizontal locomotor activity independently of task-specific locomotor proxy measures (Fig. 1b). These chambers can be adapted to deliver 20-mg reward pellets via a dispenser positioned above the reward collection magazine, but the use of this type of reinforcement was not assessed in the current study (Fig. 1a).

For both the PR and ERC paradigms, a black Perspex mask was placed in front of the touchscreen to guide responding and minimise unintended screen contact by the mice. For these paradigms, the standard ‘5-choice’ mask (Campden Instruments Ltd) was used; this consists of a row of five square apertures of 4 × 4 cm each spaced 1 cm apart across the mask positioned 1.5 cm above the floor of the arena (Fig. 1b). In both paradigms, the central response aperture was the only location in which stimuli were ever presented so as to mirror as closely as possible non-touchscreen versions of these tasks, in which the location of the manipulandum is fixed. The mask was used in every behavioural session to avoid potential neophobia resulting from its removal destabilising task performance. This mask configuration was selected in favour of a two-location variant to maximise spatial location capacity for future task developments.

### Touchscreen behavioural chamber training

Animals were habituated to the behavioural chambers for two consecutive 20-min sessions in which IR beam breaks at the touchscreen, in the magazine and across the floor of the arena were recorded, with no programmed consequences for these responses (Fig. 1c). To aid habituation, 200 μL of milkshake was delivered to the magazine at the beginning of each session; all mice consumed the reward in both sessions.

Initial operant training commenced with a 60-min session to associate touchscreen visual stimulus offset with reward delivery. This consisted of presentation of a visual stimulus (a white square) in the central screen aperture for 30 s. Upon stimulus offset, a tone (1000 ms, 3 kHz) was issued, the magazine was illuminated and 20 μL of reward delivered. Upon reward collection, the magazine light was turned off and a 5-s inter-trial interval (ITI) followed. A new trial then began. If the mouse touched the stimulus location while illuminated, the stimulus was immediately turned off, the tone issued and the magazine illuminated. A triple delivery of reward was provided on these trials. Animals were considered successfully trained on this phase once 30 rewards were collected during a session (Fig. 1c).

### Fixed and progressive ratio (FR/PR) task training

Following initial training, animals progressed to fixed ratio (FR) training. This consisted of consecutive 60-min sessions in which the central response aperture was illuminated with a white square; touching the square resulted in stimulus removal, tone delivery, magazine illumination and 20 μL reward delivery. To promote rapid responding, the ITI was reduced to 4.5 s and this was maintained for all subsequent sessions. All animals were trained on FR 1 (in which a single touchscreen response was required to earn a single reward), followed by FR 2 and FR 3 sessions (Fig. 1c). The introduction of the FR 2 and FR 3 requirements ensured animals could be trained to respond repetitively at the same touchscreen location to earn a single reward, which has not been previously demonstrated in this apparatus. Touching the stimulus in the FR 2 or FR 3 schedules resulted in brief (500 ms) removal of the screen stimulus and delivery of a ‘chirp’ tone (10 ms, 3 kHz). Criterion was defined as completion of 30 trials in a single session.

Once criterion was reached, mice progressed to the more strenuous FR 5 schedule. This phase of training used the same parameters as the earlier fixed ratio sessions and was given for three sessions to ensure animals developed high selectivity for the target location, avoiding excessive responding in the other four never-illuminated screen locations (Fig. 1c).

Following FR 5 training, animals progressed to the PR schedule. The task parameters used were identical to those used in the FR 5 sessions, except that upon completion of each trial the reward response requirement was incremented on a linear +4 basis (i.e. 1, 5, 9, 13 etc.) and if no screen response was made in the presence of a stimulus or no magazine entry was detected in the presence of delivered reward for 5 min, the session ended and the animal was removed from the chamber (Fig. 1c).

Task performance was assessed by determination of breakpoint, operationally defined as the number of target location responses emitted by an animal in the last successfully completed trial of a session. Other evaluated performance parameters included reward collection latency which was defined as the time between the completion of the final target touch of a trial and entry to the reward magazine for milkshake collection, post-reinforcement pause, defined as the interval between exit from the magazine following reward delivery and the first target touch of the next trial, total response time (TRT), defined as the time elapsed between the first and last required touchscreen response of a given trial and inter-response interval (IRI), defined as the time between each required touchscreen response of a given trial. Further variables analysed included the rate of IR beam breaks at the two arena-spanning locations (Fig. 1b) and in the reward collection magazine and the rate of touches to the four non-target touchscreen locations. Upon attainment of stable breakpoint performance, mice were systemically administered compounds to assess the effects of pharmacological manipulation on task performance (Fig. 1c).

### Effort-related choice (ERC) training

Animals were trained on FR 16, 32, and 40 for eight consecutive sessions each using the task parameters detailed previously, with the exception that three pellets of standard laboratory chow were weighed and then randomly scattered across the floor of each behavioural chamber prior to the start of each session (Fig. 1c). Upon session completion (limited to 60 min or consumption of 30 liquid rewards), mice were removed from the chambers and any remaining chow and partially eaten chow pellet fragments left on the floor of the arena, in the magazine or that had fallen into the waste collection tray were collected and weighed. Operant performance in this paradigm was evaluated in terms of the volume of milkshake consumed by an animal in a session, which is linearly related to the number of trials completed.

### Chow versus milkshake preference assessment

To ensure that C57Bl/6 mice assigned different relative values to the reward options available in the ERC paradigm in the absence of differential effort expenditure requirements, a free access home cage preference assessment was performed (Fig. 1c). This procedure was conducted in clean standard housing cages in a quiet testing room. Each cage was equipped with a small bowl that was fixed centrally to the floor. Mice were habituated to this apparatus for two consecutive 60-min sessions and then received a probe session. In the probe session, the bowl was filled with either milkshake reward or tap water, and weighed. Four standard laboratory chow pellets were also weighed and randomly placed on the floor of the cages, and mice were allowed 60 min to freely consume either substance. Upon session completion, mice were removed and returned to their home cages and the remaining chow and the bowl with liquid contents were weighed to measure consumption. This sequence was repeated for 2 weeks with the order of liquid placed in the bowl counterbalanced across the group.

### Drugs

All drugs were administered via intraperitoneal injection in a volume of 10 mL/kg. d-Amphetamine sulphate (Sigma Aldrich, Dorset, UK) was administered in a 0.9 % saline vehicle and S(−)-sulpiride (Sigma Aldrich, Dorset, UK) and S(−)-raclopride (Tocris, Bristol, UK) were administered in an acidified 0.9 % saline vehicle (final pH adjusted to 7.0 with 0.1 M NaOH). Following administration, animals were returned to their home cages for 20 or 40 min (see ‘Results’ section) prior to testing. A minimum wash out period of 11 days between each compound was used to ensure no carry-over effects occurred.

### Statistical analysis

Behavioural data were analysed by repeated-measures ANOVA with the Huynh-Feldt correction applied as determined by Mauchly’s test of sphericity or paired-sample *t* tests as appropriate. Where necessary, post hoc analysis was completed using the Bonferroni procedure to account for multiple comparisons. All analyses were conducted using SPSS 20.0 (IBM Corp., Armonk, NY, USA) with a significance level of *p* <0.05. All data are presented as mean ± standard error of the mean.

## Results

### C57Bl/6 mice can emit sustained responses to a single touchscreen location and stably maintain this behaviour

Following chamber habituation (two sessions) and initial touchscreen training (one session), all mice reached performance criterion (30 trials completed) on the FR1, FR2 and FR3 schedules in single sessions. Criterion responding on these schedules represents the emission of 30, 60 and 90 responses to the same touchscreen location with an identical visual target stimulus presented on each occasion, thereby demonstrating the viability of the touchscreen as an operant manipulandum for sustained responding equivalent to the rodent lever mechanism or nose poke aperture. Use of the more strenuous FR5 schedule, in which 150 responses were required to reach performance criterion, was also tolerated, with all animals completing 30 trials in each of the three sessions. By the third FR5 session, animals also demonstrated a significant preference for interacting with the touchscreen at the illuminated target location relative to the other four available response windows which were never illuminated (mean target/blank location response ratio = 10.2 ± 2.1:1). This compares favourably to the 3:1 discrimination ratio for the active versus inactive manipulanda criterion applied in mouse FR/PR tasks in which levers are used (Sharma et al. 2012).

**Fig. 2:**
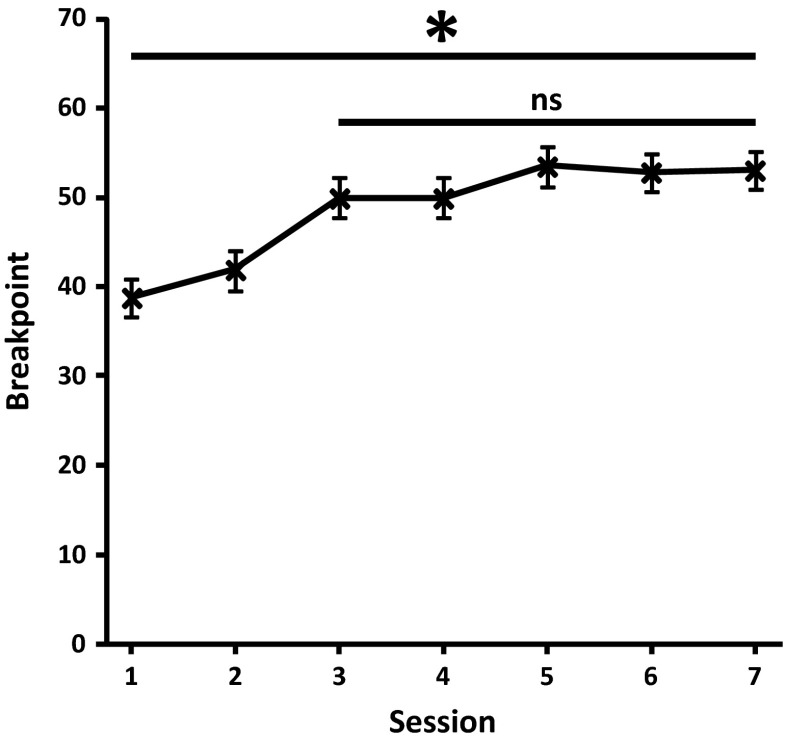
Rapid stabilisation of PR performance. Breakpoint values of C57Bl/6 mice (*n* = 18) across first seven sessions of PR4 schedule with stable breakpoint performance across final five sessions. (*p < 0.05; *ns* not significant)

Upon transfer to the linear PR4 schedule, the behavioural performance of all mice as measured by breakpoint significantly increased across the first seven sessions (*F*(2.63,44.68) = 7.34; *p* = 0.01; partial eta squared = 0.302). Post hoc analysis indicated that behavioural performance stabilised, with no significant differences (*p* = 1.000 in all cases) detected after the first three sessions (Fig. 2) (session 3 = 49.889 ± 5.417; session 4 = 49.889 ± 4.748; session 5 = 53.444 ± 4.514; session 6 = 52.778 ± 4.544; session 7 = 53.000 ± 5.224), at which point pharmacological validation could commence. A total of 16 training sessions were required to prepare naïve animals for this phase of the study.

### Touchscreen PR task performance can be enhanced by systemic d-amphetamine administration

**Fig. 3:**
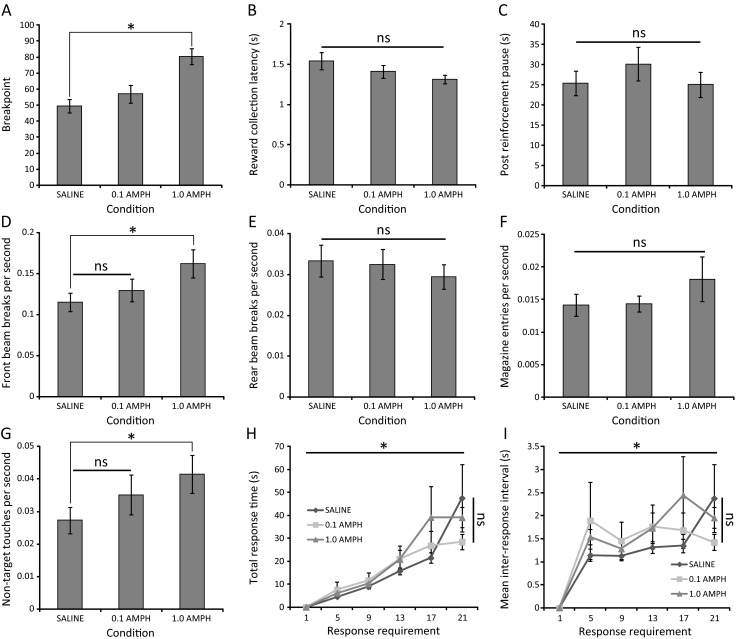
Enhancement of PR performance following systemic amphetamine administration in the absence of generalised locomotor hyperactivity. **a** Breakpoint values of C57Bl/6 mice (*n* = 18) administered 0.1 or 1.0 mg/kg d-amphetamine versus vehicle. **b** Reward collection latency and **c** post-reinforcement pause are unaffected by amphetamine administration. **d**–**f** Amphetamine differentially affects IR beam break rates in the front and rear zones of the behavioural arena and the magazine. **g** Amphetamine increases the rate of touches at non-target response locations. **h** Amphetamine has no effect on total response time. **i** Amphetamine has no effect on inter-response interval (*p < 0.05; *ns* not significant)

Systemic administration of d-amphetamine 20 min prior to behavioural testing was found to significantly affect task performance as measured by breakpoint (*F*(2,34) = 27.40; *p* < 0.001; partial eta squared = 0.617). Post hoc analysis indicated that a significant breakpoint elevation occurred following administration of 1.0 mg/kg amphetamine, relative to saline administration (saline = 49.444 ± 4.240; 1.0 mg/kg amphetamine = 80.556 ± 4.967; *p* < 0.001) (Fig. 3a).

Critically, the amphetamine-mediated elevation in task performance did not appear to be the result of non-specific psychostimulant-induced locomotor hyperactivity as no significant effects of amphetamine administration were detected on the locomotion-related measures of reward collection latency (*F*(1.53,26.08) = 2.33; *p* = 0.127; partial eta squared = 0.121) (Fig. 3b) and post-reinforcement pause (*F*(2,34) = 1.25; *p* = 0.299; partial eta squared = 0.068) (Fig. 3c).

This finding is supported by analysis of the rate of IR beam breaks across the floor of the arena and in the reward collection magazine (Fig. 3d–f). Specifically, no effect of amphetamine administration was detected on the rate of IR beam breaks in the rear zone of the chamber (*F*(2,34) = 0.915; *p* = 0.410; partial eta squared = 0.051) or the magazine (*F*(1.592,27.07) = 1.13; *p* = 0.327; partial eta squared = 0.062). In contrast, significant effects of amphetamine administration were detected on the IR beam break rate in the front zone of the chamber (*F*(1.578,26.824) = 10.37; *p* < 0.001; partial eta squared = 0.379) with a significant increase detected after 1.0 mg/kg amphetamine administration (saline = 0.115 ± 0.011; 1.0 mg/kg amphetamine = 0.163 ± 0.017; *p* = 0.006) (Fig. 3d–f). This is suggestive of enhanced motivation to ambulate towards and remain in the vicinity of the screen to enable interaction and facilitate task performance.

Enhanced activity at the screen is further supported by an effect of amphetamine administration of the rate of screen touches at non-target locations (*F*(2,34) = 3.585; *p* = 0.039; partial eta squared = 0.174). Post hoc analysis indicates a significant increase in the rate of such responses only after 1.0 mg/kg amphetamine administration (saline = 0.027 ± 0.004; 1.0 mg/kg amphetamine = 0.041 ± 0.006; *p* = 0.030) (Fig. 3g). It should be noted that, unlike paradigms in which a single ‘inactive’ manipulandum in a distinct spatial location is used to provide an estimate of non-specific activity relative to a single ‘active’ manipulandum in another location (Olausson et al. 2013), the touchscreen approach provides four ‘non-target’ locations in a relatively continuous array centred around the single ‘target’ location. The minimum proximity of ‘non-target’ locations to the single ‘target’ location is therefore considerably smaller in the touchscreen and it is therefore more likely that ‘non-target’ responses will be made in this paradigm, despite mice exhibiting high target location specificity during training. Therefore, an increase in the rate of ‘non-target’ touches in the touchscreen paradigm following amphetamine administration is arguably more representative of a highly motivated animal emitting ‘near miss’ responses at array locations adjacent to the ‘target’ location.

Analysis of the topography of responding following amphetamine administration indicated that while a main effect of response requirement was detected in both total response time and inter-response interval (TRT—*F*(2.624,44.608) = 23.267; *p* < 0.001; partial eta squared = 0.578; IRI—*F*(4.145,70.458) = 12.745; *p* < 0.001; partial eta squared = 0.428), no effect of amphetamine (TRT—*F*(2,34) = 0.383; *p* = 0.685; partial eta squared = 0.022; IRI—*F*(1.607, 27.315) = 0.335; *p* = 0.671; partial eta squared = 0.019) or a response requirement × amphetamine interaction (TRT—*F*(3.010,51.170) = 1.331; *p* = 0.275; partial eta squared = 0.073; IRI—*F*(4.454,75.717) = 1.207; *p* = 0.315; partial eta squared = 0.066) was apparent in either measure (Fig. 3h, i).

### Touchscreen PR task performance can be impaired by systemic D2 receptor antagonist administration

To assess the bidirectional sensitivity of touchscreen PR performance to pharmacological manipulation, the D2 receptor antagonist sulpiride was administered as this compound decreases breakpoint in non-touchscreen versions of the paradigm (Yoneda et al. 2007; Sakamoto et al. 2015). Initially, the same 20-min post-injection delay as used for amphetamine administration was applied; however, sulpiride administration did not affect breakpoint across a wide range of doses up to and including 100 mg/kg (data not shown).

While breakpoint remained unchanged, sulpiride administration did affect IR beam break rates, with a marginally non-significant decrease detected in the front zone of the chamber following administration of 10 mg/kg (main effect—*F*(2,34) = 3.82; *p* = 0.032; partial eta squared = 0.183; post hoc analysis (saline = 0.160 ± 0.014; 10 mg/kg sulpiride = 0.134 ± 0.011; *p* = 0.057)). Beam break rate in both front and rear zones was significantly affected at 100 mg/kg (*t*(17) = 4.14; *p* = 0.001 (saline = 0.14 ± 0.018; 100 mg/kg sulpiride = 0.10 ± 0.012) and *t*(17) = 2.38; *p* = 0.029 (saline = 0.039 ± 0.004; 100 mg/kg sulpiride = 0.028 ± 0.003), respectively), suggestive of some pharmacological activity (data not shown). In contrast, no effect of these doses was detected on the other locomotor-related measures reward collection latency (vehicle vs. 7.5 vs. 10 mg/kg (*F*(1.54,26.25) = 1.05; *p* = 0.347; partial eta squared = 0.058)), vehicle vs. 100 mg/kg (*t*(17) = −1.69; *p* = 0.110)) or post-reinforcement pause (vehicle vs. 7.5 vs.10 mg/kg (*F*(2,34) = 0.89; *p* = 0.419; partial eta squared = 0.050), vehicle vs. 100 mg/kg (*t*(17) = 0.66; *p* = 0.517)), suggestive of a potential dissociation between task-related and task-independent locomotor activity measures.

**Fig. 4:**
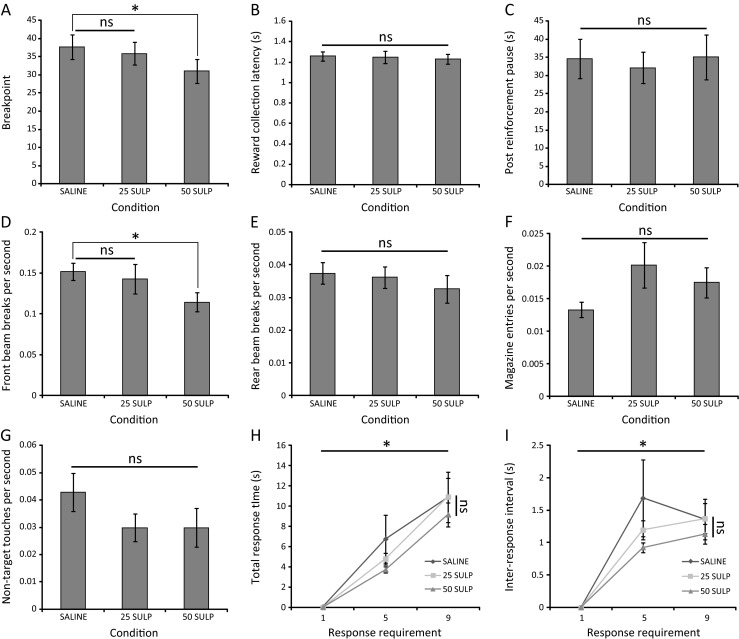
Suppression of PR performance following systemic sulpiride administration with a 40-min injection-behavioural test interval. **a** Breakpoint values of C57Bl/6 mice (*n* = 18) administered 25 or 50 mg/kg sulpiride. **b**–**c** Reward collection latency and post-reinforcement pause are unaffected by sulpiride administration. **d**–**f** Differential effects of sulpiride on the rates of IR beam breaks in the behavioural arena and magazine. **g** Effects of sulpiride on the rate of responding at non-target touchscreen locations. **h** Sulpiride has no effect on total response time. **i** Sulpiride has no effect on inter-response interval. (*p < 0.05; *ns* not significant)

Due to the lack of sulpiride effect on task performance in the prior experiments, a subset of doses were repeated with a 40-min interval between administration and behavioural testing as this delay has been used in the administration of sulpiride in other behavioural paradigms (Costall and Naylor 1995, 1997). While no effect of sulpiride on task performance as measured by breakpoint was detected following administration of either 5 or 10 mg/kg (*F*(2,34) = 1.39; *p* = 0.264; partial eta squared = 0.075) (data not shown), a significant effect was detected following administration of 25 or 50 mg/kg (*F*(2,34) = 3.98; *p* = 0.028; partial eta squared = 0.190) with post hoc analysis indicating a significant decrease in breakpoint following 50 mg/kg sulpiride relative to vehicle (saline = 37.67 ± 3.441; 50 mg/kg sulpiride = 31.00 ± 3.238; *p* = 0.009) (Fig. 4a).

Administration of 25 and 50 mg/kg sulpiride with a 40-min delay prior to behavioural evaluation was not found to significantly affect reward collection latency (*F*(2,34) = 0.15; *p* = 0.860; partial eta squared = 0.009) (Fig. 4b) or post-reinforcement pause (*F*(2,34) = 0.10; *p* = 0.909; partial eta squared = 0.006) (Fig. 4c). Sulpiride also had no effect on IR beam break rate in the rear zone of the chamber (*F*(2,34) = 0.882; *p* = 0.423; partial eta squared = 0.049) (Fig. 4d–f).

In contrast, sulpiride had a marginally significant effect on the magazine IR beam break rate (*F*(2,34) = 3.32; *p* = 0.048; partial eta squared = 0.163); however, post hoc analysis revealed no significant differences between the drug conditions (saline = 0.0133 ± 0.00491; 25 mg/kg sulpiride = 0.0201 ± 0.01486; 50 mg/kg sulpiride = 0.0175 ± 0.00979; *p* > 0.05 for all comparisons). A main effect of sulpiride administration was detected on the rate of IR beam breaks in the front zone of the chamber (*F*(1.33,22.66) = 5.07; *p* = 0.026; partial eta squared = 0.230) with post hoc analysis indicating a significant decrease relative to vehicle in the 50 mg/kg condition (saline = 0.151 ± 0.011; 50 mg/kg sulpiride = 0.114 ± 0.011; *p* = 0.008), suggestive of reduced activity proximal to the touchscreen (Fig. 4d–f). In addition, a marginally non-significant main effect of sulpiride administration was also detected on the rate of non-target location touches (*F*(2,34) = 3.251; *p* = 0.051; partial eta squared = 0.161), indicative of reduced activity at the touchscreen (Fig. 4g).

Response topography analysis also detected a main effect of response requirement on both total response time and inter-response interval (TRT—*F*(2,34) = 67.642; *p* < 0.001; partial eta squared = 0.799; IRI—*F*(1.612,27.402) = 38.798; *p* < 0.001; partial eta squared = 0.695). However, no effect of sulpiride administration (TRT—*F*(1.317,22.393) = 0.595; *p* = 0.493; partial eta squared = 0.034; IRI—*F*(1.180,20.057) = 0.799; *p* = 0.402; partial eta squared = 0.045) or a response requirement × sulpiride interaction (TRT—*F*(2.589,44.015) = 0.551; *p* = 0.625; partial eta squared = 0.031; IRI—*F*(2.000,33.994) = 1.031; *p* = 0.368; partial eta squared = 0.057) was detected in either measure (Fig. 4h, i).

**Fig. 5:**
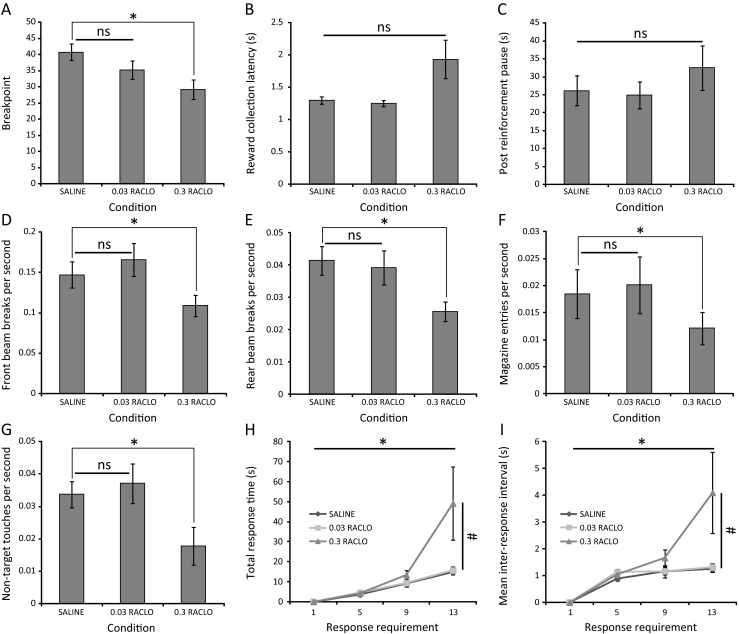
Suppression of PR performance following systemic raclopride administration. **a** Breakpoint values of C57Bl/6 mice (*n* = 18) administered 0.03 or 0.3 mg/kg raclopride versus vehicle. **b**–**c** Raclopride differentially affects reward collection latency and post reinforcement pause. **d**–**f** Effect of raclopride on the rate of IR beam breaks in the behavioural arena and magazine. **g** Effect of raclopride on the rate of responding at non-target touchscreen locations. **h** Effects of raclopride on total response time across multiple response requirements. **i** Effects of raclopride on inter-response interval across multiple response requirements (*p < 0.05; #*p* < 0.08; *ns* not significant)

Following the sulpiride validation study, the D2 receptor antagonist raclopride was selected to further validate the paradigm as this compound has similarly been reported to suppress breakpoint in non-touchscreen versions of the PR task (Cheeta et al. 1995; Aberman et al. 1998; Hajnal et al. 2007). Raclopride administration 20 min prior to behavioural testing was found to significantly affect task performance as measured by breakpoint (*F*(2,34) = 11.67; *p* < 0.001; partial eta squared = 0.407). Post hoc analysis indicated that administration of this compound at 0.3 mg/kg significantly reduced the breakpoint achieved relative to vehicle (saline = 40.778 ± 2.52; 0.3 mg/kg raclopride = 29.222 ± 3.024; *p* = 0.001) (Fig. 5a).

While no effect of raclopride administration was detected on the post-reinforcement pause measure (*F*(2,34) = 0.85; *p* = 0.438; partial eta squared = 0.047) (Fig. 5b), non-specific suppression of locomotor activity may have contributed to the observed breakpoint reduction as a significant change in reward collection latency was observed following raclopride administration (*F*(1.04,17.61) = 6.08; *p* = 0.023; partial eta squared = 0.263) although post hoc analysis indicated no significant differences between either dose of raclopride and saline (Fig. 5c). Similar main effects of raclopride administration were detected on the IR beam break rate in the front (*F*(2,34) = 12.696; *p* < 0.001; partial eta squared = 0.428) and rear zones of the chamber (*F*(2,34) = 6.862; *p* = 0.003; partial eta squared = 0.288) and in the magazine (*F*(1.581,26.880) = 6.755; *p* = 0.007; partial eta squared = 0.284) with post hoc analysis indicating a significant decrease from vehicle only in the 0.3 mg/kg raclopride condition (front beam break rate—saline = 0.147 ± 0.016; 0.3 mg/kg raclopride = 0.109 ± 0.013; *p* = 0.010; rear beam break rate—saline = 0.041 ± 0.004; 0.3 mg/kg raclopride = 0.026 ± 0.003; *p* = 0.001; magazine rate—saline = 0.018 ± 0.002; 0.3 mg/kg raclopride = 0.012 ± 0.001; *p* = 0.001) in all cases (Fig. 5d–f). A main effect of raclopride was also detected on the rate of non-target location touches *F*(2,34) = 7.177; *p* = 0.003; partial eta squared = 0.297) with post hoc analysis indicating a significant decrease following 0.3 mg/kg raclopride administration (saline = 0.0337 ± 0.03056; 0.3 mg/kg raclopride = 0.0179 ± 0.01796; *p* = 0.005) (Fig. 5g).

Response topography analysis detected a main effect of response requirement on total response time and inter-response interval (TRT—*F*(1.051,17.865) = 14.687; *p* = 0.001; partial eta squared = 0.464; IRI—*F*(1.131,19.224) = 12.787; *p* = 0.001; partial eta squared = 0.429). A trend-level main effect of raclopride (TRT—*F*(1.033, 17.566) = 3.467; *p* = 0.078; partial eta squared = 0.169; IRI—*F*(1.043,17.739) = 3.469; *p* = 0.078; partial eta squared = 0.169) and a trend-level response requirement × raclopride interaction (TRT—*F*(1.088,18.489) = 3.384; *p* = 0.079; partial eta squared = 0.166; IRI—*F*(1.182,20.089) = 3.259; *p* = 0.080; partial eta squared = 0.161) were also detected in both measures (Fig. 5h, i).

### Cost/benefit decision making in the touchscreen ERC task can be modulated by operant work requirement

Performance in the ERC task, in which rodents are required to choose between expending physical effort (i.e. nose poking or lever pressing) to obtain a highly preferred food or expending much less effort to consume a relatively less preferred food, can be varied by modifying the amount of effort required to obtain the highly preferred reward. With a minimal work requirement, rodents will favour operant responding over less preferred food consumption; however, this behaviour increasingly shifts towards the less preferred option as the operant work requirement is increased (Salamone et al. 1997).

**Fig. 6:**
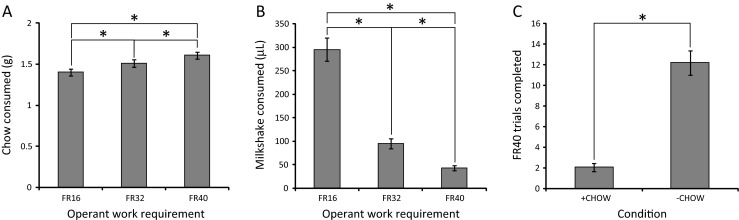
Successful training and behavioural modulation of the ERC task in the touchscreen apparatus. **a** Chow consumption across increasing operant work requirements. **b** Milkshake consumption across increasing operant work requirements. **c** Enhancement of operant responding following extended exposure to the FR40 versus chow schedule by removal of chow (*p < 0.05; *ns* not significant) (*n* = 16 for (**a**) and (**b**); *n* = 15 for (**c**))

To validate the touchscreen version of this paradigm in mice, we compared the volume of milkshake earned as a measure of the magnitude of operant responding to the amount of freely available chow consumed across the FR16, FR32 and FR40 fixed ratio work schedules. A significant effect of work requirement was detected on chow consumption (*F*(2,30) = 57.138; *p* < 0.001; partial eta squared = 0.792) with post hoc analysis indicating significant increases in consumption between each work requirement (FR16 = 1.401 ± 0.044; FR32 = 1.510 ± 0.046; FR40 = 1.607 ± 0.042; *p* < 0.001 for all comparisons) (Fig. 6a). A significant effect of work requirement was also detected on the volume of milkshake earned (*F*(1.113,16.702) = 133.187; *p* < 0.001; partial eta squared = 0.899) with post hoc analysis indicating significant decreases in milkshake volume between each work requirement (FR16 = 295.357 ± 24.841; FR32 = 95.387 ± 10.758; FR40 = 42.440 ± 5.368; *p* < 0.001 for all comparisons) (Fig. 6b). These data replicate the findings obtained in rats using a lever press variant of this task (Salamone et al. 1997).

As the FR40 schedule is highly strenuous, we were concerned that the low levels of operant responding observed in this phase of the ERC validation study were potentially due to extinction-like effects developed across the FR40 sessions. To assess this possibility, animals were given a session of the FR40 work requirement in which no laboratory chow was available in the behavioural chamber. Removal of chow from the behavioural chamber resulted in a significant enhancement in operant performance as measured by volume of milkshake earned (*t*(14) = −9.330; *p* < 0.001) (Fig. 6c), suggesting that the low levels of operant responding observed previously were primarily due to active decision-making by the animals.

### C57Bl/6 mice exhibit a substantial preference for milkshake reward relative to laboratory chow

It was important to confirm empirically that C57Bl/6 mice assigned a higher value to the reward (strawberry milkshake) obtained through operant responding in the touchscreen ERC paradigm relative to the freely available alternative (standard laboratory chow). Therefore, we presented mice with standard laboratory chow and either a sample of strawberry milkshake or water and measured consumption in each condition in a context in which effort expenditure was not required.

**Fig. 7:**
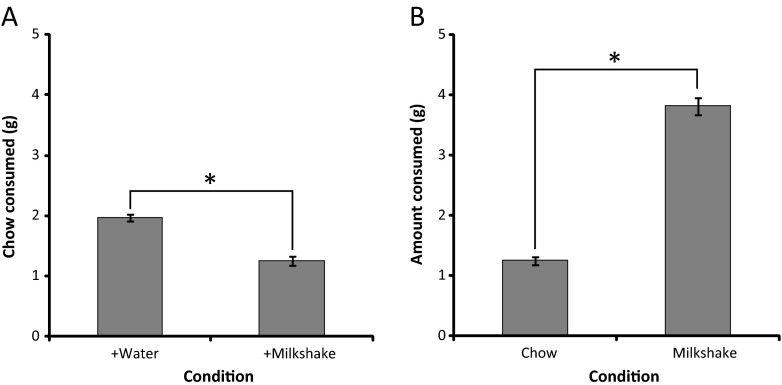
The strawberry milkshake reward used in the ERC paradigm is preferred to the standard laboratory chow alternative by C57Bl/6 mice (*n* = 15). **a** Chow consumed in the chow and water versus chow and milkshake condition. **b** Comparison of milkshake and chow consumption when both are freely available. *p < 0.05; *ns* not significant

Mice were found to consume significantly less chow when presented with chow and milkshake relative to when given the choice between chow and water (*t*(14) = 15.659; *p* < 0.001) (Fig. 7a). Also, a significantly greater amount of milkshake was consumed relative to chow in the milkshake versus chow condition (*t*(14) = −2.651; *p* = 0.019) (Fig. 7b). These data indicate that C57Bl/6 mice prefer the strawberry milkshake reward to the standard laboratory chow alternative available in the touchscreen ERC paradigm when both are freely available.

## Discussion

In this study, we report that generation and maintenance of repetitive responding at an invariant location can be supported by the rodent touchscreen system, in the context of the successful adaptation and validation of the PR (Hodos 1961) and ERC (Salamone et al. 1991) paradigms for this apparatus. These paradigms join an increasing number of tasks originally developed in operant chambers equipped with nose poke apertures or response levers that have been adapted for use in the touchscreen system and are generating similar behavioural profiles such as the 5-choice serial reaction time task (Carli et al. 1983; Romberg et al. 2011), the delayed (non)-matching to position task (Dunnett 1985; Chudasama and Muir 1997; Talpos et al. 2010) and the visuomotor conditional learning task (Reading et al. 1991; Bussey et al. 1996; Delotterie et al. 2014, 2015).

The successful adaptation of these paradigms indicates that despite the traditional rodent lever and nose poke aperture offering more extensive opportunities for sensory feedback through diverse physical interactions (e.g. snout touch, press, grip, chew etc.) and requiring an arguably more distinct motoric output to successfully register a response, the touchscreen shares similar manipulandum-like properties with these apparatus and is able to support high levels of vigorous, repetitive responding.

Further evidence for equivalence between the lever/nose poke and touchscreen versions of the PR and ERC paradigms is apparent in the pharmacological and behavioural validation studies presented here. For example, the finding that administration of amphetamine significantly increased PR performance without inducing significant non-specific locomotor hyperactivity is consistent with independent studies using a mouse nose poke version of the paradigm (Bensadoun et al. 2004) and lever-press-based versions in rats (Poncelet et al. 1983; Wirtshafter and Stratford 2010).

Indeed, the substantial increase in breakpoint (approximately 31 responses/63 % increase) induced by amphetamine administration observed in the touchscreen paradigm appears to magnify the increase observed in the nose poke variant using the same drug dose (approximately ten responses/33 % increase) (Bensadoun et al. 2004). This could suggest that the touchscreen paradigm is more sensitive to the effects of PR performance enhancers, potentially due to each touchscreen response being relatively less effortful than the equivalent nose poke entry, therefore enabling animals to perform additional trials and achieve a higher breakpoint.

Similarly, the decrements in performance generated by administration of the dopamine D2 receptor antagonists sulpiride and raclopride are congruent with data derived from non-touchscreen versions of the paradigm (Cheeta et al. 1995; Aberman et al. 1998; Hajnal et al. 2007; Yoneda et al. 2007; Sakamoto et al. 2015).

The reduction in breakpoint observed following 50 mg/kg sulpiride administration in the touchscreen paradigm (approximately six responses/18.5 % decrease) is relatively smaller than the effects reported in mice at the same dose using a lever-press variant of the task (approximately a 50 % decrease) (Sakamoto et al. 2015). In contrast to the magnified response to amphetamine, this could suggest that the touchscreen paradigm is relatively less sensitive to the effects of PR performance suppression, arguably due to the relatively lower effort expenditure required to emit a response allowing animals to extend their performance window further in the touchscreen. However, this conclusion should be tempered by the lack of a significant effect on performance following 50 mg/kg sulpiride administration in an earlier lever press PR study which represented an approximately 21 % decrease in performance (Yoneda et al. 2007). While this effect is more comparable to the magnitude of decrease observed in the touchscreen paradigm, the lack of a statistically significant effect could indicate higher behavioural variability in this version of the task (Yoneda et al. 2007) thereby effectively reducing detection sensitivity.

Like sulpiride, the reduction in breakpoint observed following administration of 0.3 mg/kg raclopride (approximately 12 responses/28 % decrease) is somewhat smaller than the effects observed in other studies using raclopride which range from approximately 40 to 90 % decreases in rat lever press (Aberman et al. 1998) and retractable sipper (Hajnal et al. 2007)-based versions of the paradigm. While this similarly supports a potentially reduced sensitivity of the touchscreen paradigm to manipulations intended to suppress PR performance, that a statistically significant effect was detectable even with a reduced absolute magnitude of suppression here highlights the low inherent variability in performance of the touchscreen paradigm.

Regarding the ERC paradigm, the finding that increasing the operant response requirement to obtain access to the preferred reward results in a shift in behaviour away from operant responding towards consumption of the freely available less palatable chow is qualitatively identical to the response shift observed in rats trained on a lever press version of this paradigm (Salamone et al. 1997). In the mouse touchscreen ERC paradigm presented here, this shift is manifested as a 14.3 % increase in chow intake and an 85.6 % decrease in milkshake consumption. In comparison, the analogous behavioural shift in the rat lever press ERC variant consists of an approximately 153 % increase in chow consumption and a 67 % decrease in reward pellet intake (Salamone et al. 1997). The difference in the magnitude of the changes in chow/reward consumption between the two paradigms is likely attributable to the species difference, the different operant work requirements used and the use of different chow/reward substances which may have been relatively more or less preferred by the animals involved in each of the studies (Salamone et al. 1997).

Taken together, these pharmacological and behavioural manipulations demonstrate that performance in the touchscreen versions of the PR and ERC tasks can be modulated in the same way as when these paradigms are delivered in chambers equipped with conventional manipulanda. These data also suggest that the touchscreen variant of the PR paradigm may magnify the effects of manipulations intended to enhance performance and, conversely, reduce the magnitude of breakpoint suppression manipulations. This potentially makes the touchscreen variant of the task better suited for the screening of compounds designed to enhance motivation/alleviate apathy-like behaviour than other variants. However, direct comparison with non-touchscreen variants is challenging due to the use of both rats and mice and wide variation in several task parameters including response requirement increment ramp, single versus repeated requirement completion contingency, inactivity time out limit, drug administration route, post-injection delay and reward substance in the studies most analogous to those presented here (Aberman et al. 1998; Bensadoun et al. 2004; Hajnal et al. 2007; Yoneda et al. 2007; Sakamoto et al. 2015). Further studies, including a parallel evaluation of different manipulanda under the same experimental and operant schedule parameters, would be needed for a comprehensive comparison.

The pharmacological validation of the PR task also revealed a marked difference between the dopamine receptor antagonists sulpiride and raclopride. While both have been previously found to reduce performance in the PR task following systemic administration (Cheeta et al. 1995; Aberman et al. 1998; Hajnal et al. 2007; Yoneda et al. 2007; Sakamoto et al. 2015), to our knowledge this is the first instance of these compounds being evaluated in the PR task in the same study.

In contrast to previous non-touchscreen studies (Yoneda et al. 2007; Sakamoto et al. 2015), our initial experiments with sulpiride indicated that this antagonist was ineffective in the PR paradigm across a wide range of doses. While potentially due to the use of the touchscreen manipulandum, this absence of effect was more likely due to the use of a 20-min injection-test delay. This was instituted to maintain technical consistency across our pharmacological validation studies, but was shorter than the delays used in prior studies in which effects of sulpiride administration were observed (Costall and Naylor 1995, 1997; Yoneda et al. 2007; Sakamoto et al. 2015). That an effect of sulpiride on touchscreen PR performance was detected after a 40-min delay is also more consistent with insufficient central antagonist accumulation during the shorter interval rather than a specific effect of the manipulandum. Indeed, the consistency between the touchscreen and conventional manipulanda with respect to pharmacological modulation of performance is supported by the significant decrease in breakpoint observed following administration of raclopride with a 20-min post-injection delay which is consistent with non-touchscreen PR studies in which the same delay was used (Aberman et al. 1998; Hajnal et al. 2007). That raclopride was able to induce significant changes in PR performance following a delay 20 min shorter than sulpiride is suggestive of pharmacokinetic differences between the antagonists and is consistent with the lower blood–brain barrier permeability and poorer in vivo central D2 antagonist properties of sulpiride relative to raclopride (Köhler et al. 1985; Ogren et al. 1986; Nakajima 1989). While further studies are required to systematically evaluate these putative pharmacokinetic differences in the context of this task, these data emphasise the importance of pharmacokinetic considerations and the use of multiple compounds with overlapping target specificity when pharmacologically validating behavioural paradigms.

The adaptation of the PR and ERC paradigms for the touchscreen apparatus permits motivation and reward-related decision making to be assessed in the same apparatus used to examine more complex cognition in laboratory rodents. While not only facilitating the study of these behavioural domains in isolation, the use of the same apparatus enables application of a battery testing approach (Horner et al. 2013; Mar et al. 2013; Oomen et al. 2013). In such a battery, individual rodents can be sequentially assessed on a wide range of tasks and incorporation of these paradigms into the battery will allow experimentally manipulated animals to be rapidly screened for unanticipated changes in motivation and reward-related decision making prior to commencement of extended cognitive assessment. If substantial motivational changes are detected, modifications (e.g. yoking the number of trials per session between experimental groups) can then be made to planned cognitive tests to appropriately compensate for motivation-related differences between groups and prevent confounding of any cognition-related study outcomes.

A relatively recent addition to the touchscreen testing apparatus has been arena floor-spanning IR beams that allow the on-line monitoring of general activity during task performance. The value of considering general activity measures during performance of behavioural tasks such as PR with this equipment has been highlighted previously (Trent et al. 2012) and can be similarly applied here. For example, administration of amphetamine resulted in an overall increase in PR performance, without affecting reward collection latency or post-reinforcement pause while administration of sulpiride yielded suppression of performance in the absence of changes in these variables. Such findings are consistent with altered motivation in the absence of pharmacologically induced generalised hyper- or hypoactivity. Evaluation of the changes in IR beam break rates also supports this interpretation in that amphetamine and sulpiride caused changes in the rate of beam breaks in the front zone of the chamber without affecting the rear zone beam break rate. This pattern of activity is consistent with a more/less motivated animal exhibiting increased/reduced task-related activity in the vicinity of the operant response location as monitored by the front zone beam break rate and yet not exhibiting evidence of non-specific hyperactivity or sedation which would manifest as parallel changes in the rear zone beam break rate. Consideration of magazine beam break rate is also constructive in that sulpiride caused significant reductions in this parameter, suggestive of animals interacting less extensively with the reward collection location due to reduced motivation to collect and consume any rewards earned following drug administration. These findings contrast with the effects of raclopride administration which resulted in a significant breakpoint reduction in concert with reductions in reward collection latency and the rate of beam breaks in the front and rear zones of the chamber and in the magazine. Such an activity profile is arguably more suggestive of a generalised suppression of locomotion by this compound, rather than a task-specific effect of administration.

Furthermore, analysis of IR beam break rates may also provide a more sensitive means by which to detect pharmacological effects in that while sulpiride was found to have no significant effect on breakpoint when administered with a 20-min interval between injection and behavioural testing, some evidence of changes in IR beam break rates was detected in these conditions. That an effect on these parameters was apparent in the absence of effects on measures more closely associated with task performance may indicate a decoupling of movements specifically related to task performance such as magazine entries and more general motor actions such as grooming, rearing and investigating the floor and corners of the arena. These data are consistent with evidence of sulpiride-mediated locomotor suppression occurring prior to changes in performance of the active allothetic place avoidance task (Stuchlik et al. 2007). In addition, this potential dichotomy in motoric output sensitivity is supported by data indicating that extended training can cause motoric behaviours previously sensitive to dopaminergic receptor antagonist exposure to become resistant to such exposure while spontaneous locomotion and the trained motoric response when emitted in the absence of the training cue remain vulnerable to antagonist-mediated suppression (Choi et al. 2005, 2011). Specifically, as the task-related motor responses required for PR performance were extensively trained prior to drug exposure and were always measured in the presence of the cues used during training, they were therefore relatively more protected from antagonist-mediated suppression than non-task-related actions. Therefore, such non-task-related activities (as measured by IR beam break rate) were suppressed at a lower dose and/or following a shorter post-injection delay than the task-related motor outputs. Further studies involving ethogram-based video analysis of behaviour during task performance following drug administration will be required to further assess this possibility.

Taken together, this study presents the successful adaptation and validation of the PR and ERC paradigms for the rodent touchscreen testing system, showing that the touchscreen can be used to support vigorous, repetitive operant responding at a single spatial location with the same target stimulus. This finding indicates that the touchscreen has manipulandum-like properties in common with the rodent lever assembly and nose poke port and will allow any task conducted with these response devices to be adapted for use in the touchscreen. These paradigms also permit direct evaluation of motivation and reward-related decision making in this apparatus, and can be used as part of a battery approach (Horner et al. 2013) to test whether the effect of an experimental manipulation on the performance of a touchscreen cognitive task could be due to unanticipated alterations in these constructs, and/or to pre-screen animals for motivational changes. To date, our laboratory has not detected any cross-over effects from testing animals in these tasks prior to evaluation with extant touchscreen cognition assays. The pharmacological and behavioural validation experiments presented in this study provide further evidence that these tests are equivalent to versions performed using non-touchscreen equipped apparatus, suggesting that the primary neurobiological mechanisms underlying PR and ERC are the same in the lever, nose-poke and touchscreen versions of these paradigms.
